# Type 1 diabetes is associated with higher plasma levels of biomarkers of neurodegeneration and neuroinflammation

**DOI:** 10.3389/fimmu.2026.1860993

**Published:** 2026-07-22

**Authors:** Meghan E. Pauley, Christina Coughlan, Fran Dong, Brian Bucca, Bailey Tanner, Catherine Chartier-Logan, Allison L. B. Shapiro, Janet K. Snell-Bergeon

**Affiliations:** 1 Barbara Davis Center for Diabetes, School of Medicine, University of Colorado Anschutz Medical Campus, Aurora, CO, United States; 2 University of Colorado Alzheimer’s and Cognition Center, University of Colorado Anschutz Medical Campus, Aurora, CO, United States; 3 Department of Neurology, School of Medicine, University of Colorado Anschutz Medical Campus, Aurora, CO, United States; 4 Lifecourse Epidemiology of Adiposity and Diabetes Center, University of Colorado Anschutz Medical Campus, Aurora, CO, United States; 5 Section of Endocrinology, Department of Pediatrics, School of Medicine, University of Colorado Anschutz Medical Campus, Aurora, CO, United States

**Keywords:** GFAP – glial fibrillary acidic protein, neurodegeneration, neurofilament light chain (NFL), neuroinflammation, phosphorylated tau (pTau), plasma biomarkers, pTau-181, type 1 diabetes

## Abstract

**Introduction:**

Type 1 diabetes (T1D) is associated with increased risk of dementias and other neurodegenerative diseases. Plasma biomarkers of neurodegeneration and neuroinflammation hold utility in risk prediction and diagnosis of neurodegenerative disease and other neuropathology, but there remains a paucity of data in the T1D population. This study quantified plasma levels of biomarkers of neurodegeneration and neuroinflammation [phosphorylated tau-181 (pTau-181), total tau (Tau), amyloid beta 42/amyloid beta 40 (Aβ42/40), glial fibrillary acidic protein (GFAP), and neurofilament light chain (NfL)], and tested associations with T1D and related characteristics.

**Methods:**

This cross-sectional analysis of a subset of participants from the Coronary Artery Calcification in Type 1 Diabetes study included adults with and without T1D. Biomarker levels were quantified using Quanterix Simoa, then log-transformed and analyzed using linear regression models adjusted for age, body mass index (BMI), race and ethnicity, smoking status, and estimated glomerular filtration rate (eGFR). A subsequent sensitivity analysis included diabetic retinopathy (DR).

**Results:**

136 adults with T1D (n=90, age 52 years, 58% female, average HbA1c 7.7%) and without T1D (n=46, age 53 years, 57% female, average HbA1c 5.4%) were included. In fully adjusted models, T1D was associated with higher pTau-181 [estimate (pg/mL) 1.221, (95% confidence interval 1.003, 1.486) p=0.046], GFAP [1.296 (1.129, 1.489), p<0.001], and NfL [1.355 (1.162, 1.579), p<0.001], relative to adults without T1D. In the sensitivity analysis, relative to adults without T1D, T1D with moderate/severe DR was associated with higher pTau-181 [1.330 (1.063, 1.663), p=0.013], GFAP [1.369 (1.653, 1.608), p<0.001], and NfL [1.495 (1.252, 1.786), p<0.001], and T1D with none/mild DR was associated with higher GFAP [1.228 (1.046, 1.442), p=0.013] and NfL [1.235 (1.037, 1.470), p=0.018]. As compared to T1D with none/mild DR, T1D with moderate/severe DR was associated with higher NfL [1.211 (1.011, 1.451), p=0.038]. Across both models, eGFR was inversely associated with pTau-181, NfL, and Tau (p<0.05 for all).

**Discussion:**

T1D is associated with a greater burden of plasma biomarkers of neurodegeneration and neuroinflammation, and microvascular complications such as DR and impaired kidney function may contribute to this burden. Further study is needed to fully characterize the interplay between these biomarkers and neuropathology in T1D.

## 1 Introduction

A growing body of evidence demonstrates that diabetes confers deleterious impacts on the central and peripheral nervous systems and is linked to neuroinflammatory and neurodegenerative disorders, including cognitive impairment syndromes and dementias (1–7). Indeed, recent literature suggests that the chronic inflammation and immune dysregulation, impaired glucose metabolism, and insulin signaling dysfunction seen in type 2 diabetes (T2D) may play a direct role in the multifactorial pathogenesis of neurodegenerative disorders (3, 8, 9). Further, some of the leading complications of diabetes, such as diabetic retinopathy (DR), are characterized by a combination of microvascular dysfunction and neurodegenerative pathophysiology and may reflect the presence of central and/or systemic neurodegeneration (10–14). However, existing studies are largely centered around adults with T2D, and there has been limited study with inconsistent findings on the association between T1D and systemic and/or central neurodegenerative pathology (7, 15–21).

The pathophysiology underlying neurodegeneration and neuroinflammation can begin decades prior to the onset of clinical symptoms (22, 23). Advances in the field of aging provide new, less-invasive avenues for exploration of these processes, even in the earliest stages of the disease trajectory. One such method is quantification of plasma levels of biomarkers of neurodegeneration and neuroinflammation, including phosphorylated Tau-181 (pTau-181), total tau protein (Tau), and amyloid beta 42/amyloid beta 40 (Aβ42/40), glial fibrillary acidic protein (GFAP), and neurofilament light chain (NfL) protein (22, 23). Studies in older adults have shown that lower or decreasing plasma levels of Aβ42/40 and higher or increasing plasma levels of pTau-181 are associated with brain amyloid deposition, which is a hallmark feature of Alzheimer’s Disease (AD) neuropathology (22, 24–27). More recently, higher plasma levels of pTau-181 have been linked to other systemic neurodegenerative diseases (28, 29). Higher or increasing levels of Tau are associated with brain tau and amyloid pathology, as well as serving as a non-specific marker of neural damage (22, 25, 30, 31). Higher or increasing levels of GFAP are a marker of glial activation and downstream neuroinflammation (22, 31, 32), and higher or increasing plasma levels of NfL are suggestive of non-specific neural damage (22, 31, 33).

Given the paucity of data regarding the relationship between T1D and neurodegenerative pathology, the current study sought to examine the association between T1D and related characteristics and biomarkers of neurodegeneration and neuroinflammation. Here, we hypothesized that T1D would be associated with higher plasma levels of pTau-181, Tau, GFAP, and NfL, and lower plasma levels of Aβ42/40.

## 2 Materials and methods

### 2.1 Study design and participants

The current study applied a cross-sectional design using data and samples collected by The Coronary Artery Calcification in Type 1 Diabetes (CACTI) study of people with and without T1D. All study procedures were approved by the Colorado Multiple Institutional Review Board (COMIRB #97-661), and all participants provided written informed consent. Patients or the public were not involved in the design, or conduct, or reporting, or dissemination plans of this research.

The CACTI study design and population have been previously reported (34, 35). Briefly, CACTI was a prospective longitudinal cohort study that investigated diabetes-associated complications in adults with T1D. In the parent study, 1416 adults, including 652 with T1D and 764 adults without diabetes (hereafter referred to as controls), aged 19 to 56 years old, completed a baseline visit between March 2000 and April 2002. Participants completed up to three additional visits over an 18-year follow-up period. All participants with T1D were younger than 30 years of age at the time of diabetes diagnosis or had positive autoantibodies or a clinical course consistent with T1D [mean and standard deviation (SD); T1D duration 23.5 (9.0) years], were initiated on insulin therapy within one year of diabetes diagnosis, and had a minimum diabetes disease duration of 10 years at time of enrollment. Controls without diabetes had not been previously diagnosed with diabetes of any kind, had fasting blood glucose < 126 mg/dL, and were the spouses, friends, and/or neighbors of enrolled participants with T1D with similar age and sex distributions to the T1D group.

**Figure 1:**
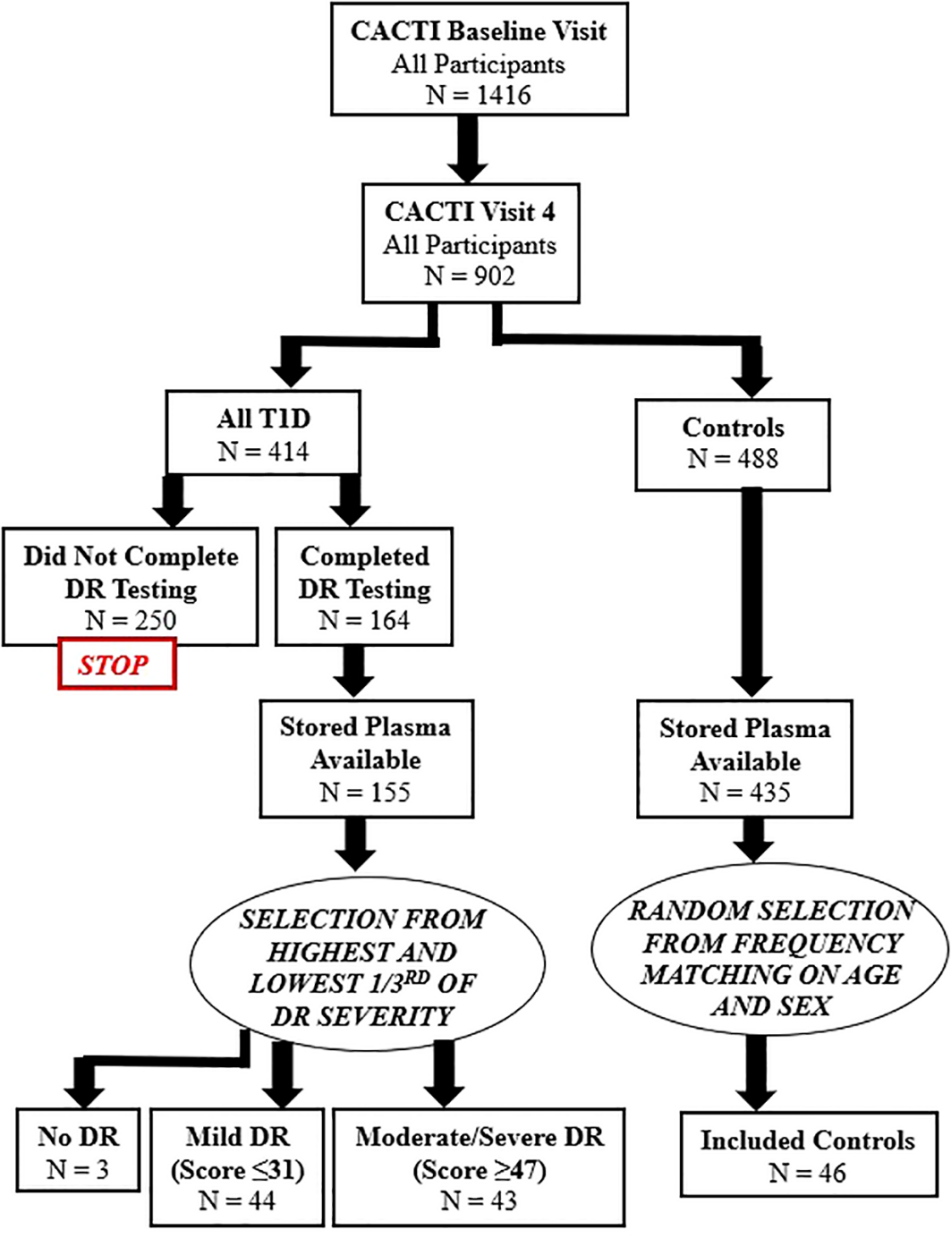
Flow diagram of participant selection. CACTI, Coronary Artery Calcification in Type 1 Diabetes study; T1D, type 1 diabetes; DR, diabetic retinopathy.

From the parent CACTI study, the current study included data and plasma specifically from the final follow-up visit, hereafter referred to as visit 4, that occurred for all participants between 2013-2018, and during which participants with T1D completed a retinal exam and venipuncture blood draw. Inclusion in the current study required completion of visit 4 procedures (all participants), stored plasma availability, and T1D duration of >15 years (T1D group only) at visit 4. Participants with T1D were selected from identifying the highest and lowest 1/3^rd^ of DR severity scores at visit 4. The T1D and control groups were frequency matched on age and sex. Controls without diabetes were excluded if they developed type 2 diabetes during the study or follow-up. Applying exclusion and matching, our analytic sample was 136 adults with and without T1D. See **Figure 1**.

### 2.2 Anthropometric and metabolic measures

Demographic information including sex and race and ethnicity was collected at baseline, and medical history was recorded at each visit during the CACTI study follow-up period, including factors such as smoking status (as “current”, “former”, or “never”). Due few participants reporting “current” smoking status (n=5), “current” and “former” smoking statuses were combined into a single group, “current/former”. At each visit, anthropometry was measured including height (m) and weight (kg), and body mass index (BMI; kg/m2) was calculated. Each visit also included an overnight fast, after which blood was collected and centrifuged, and separated plasma was stored in 0.5mL aliquots in polypropylene tubes at -80 °C in dedicated freezers at the Barbara Davis Center for Diabetes (BDC). Hemoglobin A1c (Hba1c) was quantified by high-performance liquid chromatography method (HPLC, BioRad variant), performed in the Clinical Laboratory at the University of Colorado Hospital. Estimated glomerular filtration rate (eGFR) was calculated from serum creatinine using the 2009 Chronic Kidney Disease Epidemiology Collaboration (CKD-EPI) equation.

### 2.3 Diabetic retinopathy assessment

DR was assessed at CACTI visit 4 using standard 7-field fundus photography conducted at the BDC eye clinic after dilation by addition of 1% Tropicamide and 2.5% Phenylepherine to both eyes. Color stereoscopic fundus photographs of seven standard fields in both eyes were obtained using the Early Treatment Diabetic Retinopathy Study (ETDRS) protocol and a Canon 60UVi camera. The photographs were electronically transferred to the University of Wisconsin Ocular Epidemiology Reading Center (OERC) for blind grading of severity. Due to having few participants with T1D with no DR (n=3), DR severity was collapsed into two groups: none/mild DR (score of ≤31) and moderate/severe DR (score of ≥47), in order to differentiate participants with minimal or no DR from those with clinically meaningful DR. If a participant had differing degrees of DR in each eye, DR classification was based upon the most severe degree noted.

### 2.4 Plasma biomarkers of neurodegeneration and neuroinflammation

Biomarkers were quantified by the University of Colorado Alzheimer’s and Cognition Center using the ultrasensitive Quanterix Simoa^®^ platform, including the Neurology 3-Plex A Advantage Kit measuring Aβ42, Aβ40, and Tau, the Neurology 2-Plex B Kit measuring GFAP and NfL, and the pTau-181 Advantage V2.1 Kit, according to the manufacturer’s protocols. At the time of writing, the Quanterix Simoa^®^ kits/assays used in this study are different than versions used in comparable published studies, which may limit direct comparison of raw biomarker measures.^1^ One such example is the pTau-181 Advantage V2.1 Kit, which was a 2023 update to the previous V2 Kit to further improve pTau-181 sensitivity. Samples were run in duplicate, and values were averaged across the duplicate measures. Plasma aliquots were thawed only once prior to this analysis.

### 2.5 Statistical analysis

All biomarker analyses were restricted to replicates with a coefficient of variation (CV) <20% [pTau-181 (n=14), Aβ42/Aβ40 (n=3), Tau (n=2), GFAP (n=14), NfL (n-7)]. Plasma samples were run in duplicate; in the event that only one of two sample repeats were successful, the single successful measurement was included in this analysis [Aβ42/40 (n=1), Tau (n=1), GFAP (n=6), NfL (n=6)]. There was n=1 of Aβ42/40, Tau, GFAP, and NfL, where neither sample was successful, and therefore no data quantified. Additionally, one participant’s Aβ42/40 was determined to be an outlier after evaluation with Cook’s Distance (Cook’s D Influence Statistic= 0.729), and was excluded from the models.

Descriptive characteristics are reported as means and standard deviation (SD), median and interquartile range (IQR), or frequency count and proportion, as appropriate. Of note, HbA1c was reported for descriptive purposes only and was not included as a covariate in the analyses due to the bimodal distribution, whereby participants with T1D had distinctly higher HbA1c value, as expected. Biomarker levels were log-transformed due to right-skewed distributions. Multivariable linear regression models were run for each biomarker outcome, setting T1D status (T1D vs. control) as the primary predictor variable. Each model included adjustment for relevant covariates including age (continuous), BMI (continuous), sex (female vs. male), race and ethnicity (non-Hispanic White vs. all others), smoking status (current/former vs. never), and kidney function (eGFR). Race/ethnicity was dichotomized to NHW vs. all others due to the low representation of other race/ethnicities, which would not support meaningful comparisons.

In a sensitivity analysis, we included a multilevel categorical variable for DR status to explore the influence of DR severity on the associations observed between T1D and biomarker levels. Again, a multivariable linear regression model was run including each biomarker as the outcome, setting DR status as the primary predictor (T1D with moderate/severe DR, T1D with none/mild DR, controls [reference]). Within the sensitivity models, we estimated contrasts comparing T1D with moderate/severe DR vs. controls, T1D with none/mild DR vs. controls, and T1D with moderate/severe DR vs. T1D with none/mild DR. The resultant beta coefficients from both regression models were then back-transformed and reported in the results section below. Data analyses were performed using SAS software version 9.4 (SAS Institute, Cary, NC). Two-sided P values <0.05 were considered significant.

## 3 Results

136 participants were included in this study, including n=46 controls without diabetes and n=90 with T1D. The control group had a mean age of 52.5 years, were 56.5% female, 93.5% NHW. The T1D group had a mean age of 52.0 years, were 57.8% female, 95.6% NHW, with mean HbA1c of 7.6% at CACTI visit 4, and an average HbA1c of 7.7% across all CACTI visits. Of those with T1D, n=47 had none/mild DR, and n=43 had moderate/severe DR. These two groups were relatively similar in age and HbA1cs, but had differences in T1D duration, eGFR, and sex distribution. Participant characteristics are reported by group in **Table 1**. Unadjusted biomarker medians and interquartile ranges are reported in **Supplementary Table 1**.

**Table 1:** Participant characteristics at visit 4, by group (n=136).

Characteristic	Controls n=46	All T1D n=90	T1D with None/Mild DR n=47	T1D with Moderate/Severe DR n=43
Age in years (mean ± SD)	52.5 ± 9.3	52.0 ± 9.1	52.4 ± 10.1	51.5 ± 8.1
NHW race/ethnicity (n, %)	43, 93.5%	86, 95.6%	46, 97.9%	40, 93.0%
Female sex (n, %)	26, 56.5%	52, 57.8%	24, 51.1%	28, 65.1%
T1D duration in years (mean ± SD, range)	--	37.4 ± 8.2	34.6 ± 8.0	40.4 ± 7.5
Visit 4 HbA1c% (mean ± SD)	5.4 ± 0.4	7.6 ± 1.0	7.5 ± 0.8	7.8 ± 1.1
Average HbA1c% (mean ± SD)	5.4 ± 0.3	7.7 ± 1.0	7.5 ± 0.7	7.9 ± 1.3
eGFR in mL/min/1.73 m^2^ (mean ± SD)	84.2 ± 14.2	84.7 ± 20.5	88.0 ± 17.3	81.0 ± 23.2
BMI in kg/m^2^ (mean ± SD)	27.5 ± 5.4	26.6 ± 4.4	26.4 ± 4.4	26.9 ± 4.4
Smoking status (n, %) Current/Former Never	18, 39.1% 28, 60.9%	26, 28.9% 64, 71.1%	15, 31.9% 32, 68.1%	11, 25.6% 32, 74.4%

Data presented as means and standard deviation or count and proportion, as noted. T1D, type 1 diabetes; DR, diabetic retinopathy; SD, standard deviation; BMI, body mass index; Visit 4 HbA1c, hemoglobin A1c measurement at CACTI study visit 4 only; Average HbA1c, the average of hemoglobin A1c measurements across all CACTI study visits; eGFR, estimated glomerular filtration rate (calculated per the 2009 Chronic Kidney Disease Epidemiology Collaboration equation); NHW, non-Hispanic white.

Results from the primary linear regression models are reported in **Table 2**. Independent of age, BMI, sex, race, smoking status, and eGFR, T1D was significantly associated with higher pTau-181, GFAP, and NfL plasma levels. Age was significantly associated with lower Aβ42/40 levels and higher GFAP and NfL levels. Female sex was significantly associated with lower pTau-181 levels, and BMI was significantly associated with lower Aβ42/40 levels. Smoking status and NHW race were not significantly associated with any biomarker levels. Lower eGFR was significantly associated with higher pTau-181, NfL, and Tau levels. For these biomarkers that demonstrated a significant relationship with eGFR (pTau-181, NfL, and Tau), we also conducted testing for interaction between T1D and eGFR, and none were significant (p=0.094, 0.927, and 0.389, respectively).

**Table 2:** Association between T1D status and biomarker levels, adjusted for age, sex, BMI, race, smoking status, and eGFR.

Variable	pTau-181	Aβ42/40	GFAP	NfL	Tau
All T1D vs. controls	1.221 [1.003, 1.486] **0.046**	0.983 [0.928, 1.041] 0.553	1.296 [1.129, 1.489] **<0.001**	1.355 [1.162, 1.579] **<0.001**	1.000 [0.864, 1.157] 0.998
Age	1.009 [0.997, 1.021] 0.134	0.996 [0.993, 0.999] **0.032**	1.027 [1.018, 1.037] **<0.001**	1.013 [1.003, 1.022] **0.011**	0.999 [0.990, 1.008] 0.831
Female sex	0.782 [0.643, 0.951] **0.014**	0.956 [0.901, 1.013] 0.127	1.134 [0.980, 1.312] 0.090	0.900 [0.771, 1.050] 0.179	1.053 [0.907, 1.222] 0.496
BMI	1.004 [0.985, 1.023] 0.684	0.993 [0.987, 0.999] **0.017**	0.989 [0.975, 1.003] 0.138	0.987 [0.971, 1.002] 0.096	0.995 [0.980, 1.009] 0.466
NHW race/ethnicity	0.815 [0.536, 1.238] 0.334	1.001 [0.888, 1.128] 0.988	1.078 [0.797, 1.458] 0.622	0.814 [0.579, 1.144] 0.234	1.113 [0.819, 1.515] 0.490
Smoke: current/former vs. never	0.862 [0.699, 1.063] 0.163	1.012 [0.952, 1.076] 0.692	0.946 [0.818, 1.095] 0.456	0.978 [0.834, 1.148] 0.786	1.035 [0.885, 1.209] 0.665
eGFR	0.990 [0.985, 0.996] **0.002**	0.999 [0.998, 1.001] 0.616	0.996 [0.992, 1.000] 0.075	0.990 [0.985, 0.995] **<0.001**	0.994 [0.989, 0.998] **0.008**

Data presented as back-transformed beta coefficients and 95% confidence intervals, with associated *p*-values. Significant *p*-values are bolded.

T1D, type 1 diabetes; SE, standard error; pTau-181, phosphorylated tau-181; Aβ42/40, amyloid beta 42/amyloid beta 40; GFAP, glial fibrillary acidic protein; NfL, neurofilament light chain; Tau, total tau; BMI, body mass index; NHW, non-Hispanic white; eGFR, estimated glomerular filtration rate (calculated per the 2009 Chronic Kidney Disease Epidemiology Collaboration equation).

Results from the sensitivity analysis are reported in **Table 3**. As compared to controls, T1D with moderate/severe DR was significantly associated with higher pTau-181, GFAP, and NfL levels. T1D with none/mild DR was also associated with higher GFAP and NfL, compared to controls. Finally, as compared to T1D with none/mild DR, T1D with moderate/severe DR was associated with higher NfL levels. Again, age was significantly associated with lower Aβ42/40 levels but higher GFAP and NfL levels. Female sex was significantly associated with lower pTau-181 levels, and higher BMI was significantly associated with lower Aβ42/40 levels. Smoking status and NHW race were not significantly associated with any biomarker levels. Lower eGFR was significantly associated with higher pTau-181, NfL, and Tau levels.

**Table 3:** Association among T1D status, DR severity, and biomarker levels, adjusted for age, sex, BMI, race, smoking status, and eGFR.

Variable	pTau-181	Aβ42/40	GFAP	NfL	Tau
T1D with moderate/severe DR vs. controls	1.330 [1.063, 1.663] **0.013**	0.974 [0.911, 1.042] 0.450	1.369 [1.165, 1.608] **<0.001**	1.495 [1.252, 1.786] **<0.001**	1.051 [0.885, 1.248] 0.568
T1D with none/mild DR vs. controls	1.117 [0.890, 1.401] 0.336	0.991 [0.927, 1.059] 0.782	1.228 [1.046, 1.442] **0.013**	1.235 [1.037, 1.470] **0.018**	0.956 [0.808, 1.130] 0.594
T1D with moderate/severe DR vs. T1D with none/mild DR	1.191 [0.951, 1.491] 0.127	0.984 [0.919, 1.053] 0.631	1.115 [0.945, 1.315] 0.195	1.211 [1.011, 1.451] **0.038**	1.100 [0.924, 1.308] 0.281
Age	1.010 [0.998, 1.022] 0.096	0.996 [0.992, 0.999] **0.030**	1.028 [1.019, 1.037] **<0.001**	1.014 [1.005, 1.024 **0.004**	0.999 [0.990, 1.009] 0.920
Female sex	0.774 [0.637, 0.940] **0.010**	0.956 [0.902, 1.014] 0.135	1.131 [0.978, 1.308] 0.097	0.889 [0.764, 1.036] 0.132	1.046 [0.900, 1.214] 0.556
BMI	1.003 [0.984, 1.023] 0.737	0.993 [0.987, 0.999] **0.018**	0.989 [0.975, 1.003] 0.113	0.985 [0.970, 1.001 0.060	0.994 [0.980, 1.010] 0.435
NHW race/ethnicity	0.831 [0.548, 1.261] 0.382	0.998 [0.884, 1.126] 0.970	1.092 [0.807, 1.476] 0.565	0.853 [0.608, 1.197] 0.355	1.134 [0.833, 1.545] 0.421
Smoke: current/former vs. never	0.866 [0.703, 1.067] 0.175	1.011 [0.951, 1.075] 0.724	0.954 [0.824, 1.104] 0.524	0.991 [0.846, 1.161] 0.912	1.043 [0.892, 1.219] 0.597
eGFR	0.991 [0.986, 0.997] **0.006**	0.999 [0.998, 1.001] 0.564	0.996 [0.992, 1.001] 0.122	0.991 [0.986, 0.996] **<0.001**	0.994 [0.990, 0.999] **0.015**

Data presented as back-transformed beta coefficients with 95% confidence intervals, with associated *p*-values. Significant *p*-values are bolded. T1D, type 1 diabetes; DR, diabetic retinopathy; SE, standard error; pTau-181, phosphorylated tau-181; Aβ42/40, amyloid beta 42/amyloid beta 40; GFAP, glial fibrillary acidic protein; NfL, neurofilament light chain; Tau, total tau; BMI, body mass index; NHW, non-Hispanic white; eGFR, estimated glomerular filtration rate.

Using the model residual standard deviation (Root MSE = 0.174) to standardize adjusted regression coefficients, the biomarker NfL, which exhibited the strongest association with DR severity, showed effect sizes ranging from d=0.53 for those with none/mild DR, to d=1.01 for those with moderate/severe DR, relative to controls. In contrast, the non-significant biomarker Tau demonstrated very small effects, ranging from d=-0.11 to 0.13. The sample size in our study provided over 99% power to detect the large effect size of 1.01 for the comparison of NfL between those with moderate/severe DR relative to controls. The power provided by this sample size to detect the medium effects size of 0.53 observed for the comparison of NfL in those with no/mild DR was 70%, and the power to detect the very small effects sizes observed for the group comparisons for Tau were 0.09% and 0.08%. Therefore, if the true differences between these groups are small to medium, then our study may have failed to detect significant differences due to insufficient power.

## 4 Discussion

In this study, T1D was significantly associated with higher plasma levels of biomarkers of neurodegeneration and neuroinflammation (pTau-181, GFAP, and NfL) when compared to similar aged adults without diabetes, and T1D-associated microvascular complications such as DR and impaired kidney function may contribute to these relationships.

In populations at risk of dementia, plasma levels of pTau-181 are shown to increase decades before clinical symptoms of cognitive impairment become apparent (31, 36). Plasma pTau-181 is a validated biomarker of AD neuropathology, demonstrating discriminatory power to identify presence of brain Aβ pathology, cognitive decline, and dementia in older adults (37, 38). However, while implicated in the pathology of central nervous system neurodegenerative diseases, more recently, plasma pTau-181 has also been linked to systemic neurodegenerative diseases, with speculation of a peripheral origin in amyotrophic lateral sclerosis (ALS), for example (28, 29). Thus, the specific neural origin of pTau-181 in plasma is unclear, and while our pTau-181 findings are perhaps most notable, they should be interpreted with caution. As there has been limited study of pTau-181 in those with T1D further research is needed to better understand the origin and role of pTau-181 in individuals with T1D.

Aβ are also known biomarkers of AD neuropathology in at-risk populations. However, inverse to the interpretation of pTau-181 plasma levels, decreasing plasma levels of Aβ42/40 are suggested to reflect increased brain amyloid deposition, and relate to worse cognitive function and increased risk of AD (31, 39). Yet, some studies demonstrate non-linear relationships between plasma levels of Aβ biomarkers, cognitive function, and the dementia continuum, with plasma levels sometimes measuring higher earlier in the clinical course, and then declining as disease progresses (40–42). Importantly, Aβ in plasma are not shown to perform as well as when measured in cerebral spinal fluid for discriminating AD neuropathology or cognitive impairment (31, 43). As few studies have examined plasma biomarkers of neurodegeneration and neuroinflammation in the T1D population, additional work is needed to validate our study’s findings, and fully determine the associated implications.

GFAP and NfL are suggested to be biomarkers of nonspecific neuroinflammation and neurodegeneration, respectively. Specifically, GFAP is a cytoskeletal component of astrocytes and plasma levels are shown to be elevated in the setting of reactive astrocytosis (31). NfL is an axonal scaffolding protein and core neurofilament protein in the central nervous system (31). While both GFAP and NfL demonstrate relationships with the AD neuropathological continuum in at-risk populations, they are also related to other central nervous system injuries and diseases (39, 44–46). Our results suggest that T1D is associated with higher plasma levels of GFAP and NfL, though directionality of these relationships cannot be determined due to the cross-sectional study design.

While our study is one among a small number of studies investigating relationships between T1D and biomarkers of neurodegenerative disease, our results are mostly consistent with existing evidence. In a proof-of-concept study, our team (Shapiro, et al., 2024) quantified biomarkers in a group of young adults with youth-onset T1D or T2D from the SEARCH for Diabetes in Youth (SEARCH) study, and a group of age-similar controls without diabetes (47). This study demonstrated that those with youth-onset diabetes had higher pTau-181 and NfL compared to controls, which is in alignment with this study’s results. Yet, it is important to note that the life course trajectory of the included biomarkers is not fully understood, and age and developmental stage may play a significant role in how plasma levels of these biomarkers increase or decrease relative to diabetes progression. Given the SEARCH population was much younger, we caution comparing this study’s results to the SEARCH cohort.

In another larger study using data from the DCCT/EDIC cohort, Karger, et al. evaluated relationships between these plasma biomarkers, cognitive testing, and structural brain measurements in adults with T1D with median age of 60 years (48). Authors found that higher plasma NfL levels were associated with increased predicted brain age and higher odds of impaired cognition, and higher NfL and pTau-181 levels were associated with lower psychomotor and mental efficiency. Differing from our results, DCCT/EDIC found no significant association between any biomarker and proliferative DR. They found significantly higher NfL levels in those with kidney disease, which aligns with our study’s findings. Existing literature suggests that plasma biomarker levels may be influenced by kidney function, specifically protein clearance, and thus may have decreased diagnostic performance in populations with kidney disease or altered protein clearance (49–51). This prompted inclusion of eGFR in our statistical models. Biomarker ratios, such as Aβ42/40, as well as GFAP, are suggested to be less impacted by kidney function compared to other biomarkers, and our study’s findings align with this (49, 52–54).

The Glycemic Variability and Fluctuations in Cognitive Status in Adults with T1D (GluCog) study also quantified many of these biomarkers in adults with T1D (mean age 49 years) and tested associations between biomarker levels and cognitive testing (49). GluCog found that higher levels of pTau-181 and GFAP were associated with slower responses on working memory tasks, and that higher Aβ42/40 levels (non-neuropathologic direction) were associated with better vocabulary. GluCog also found that higher pTau-181 and NfL levels were associated with higher odds of kidney disease, which aligns with our results, but they did not investigate relationships with DR.

Importantly, these three comparable studies (SEARCH, DCCT/EDIC, and GluCog) used the Quanterix Simoa platform to quantify plasma biomarker levels. However, these studies used different versions of the standard kits than those used in this study. Specifically, all three studies used the Neurology 4-Plex E Advantage Kit (measuring Aβ42, Aβ40, GFAP, and NfL), whereas our study utilized the Neurology 3-Plex A Advantage Kit (measuring Aβ42, Aβ40, and Tau) and the Neurology 2-Plex B Kit (measuring GFAP and NfL). Furthermore, the SEARCH study used a prior version of the pTau-181 assay (Advantage V2 Assay Kit) which differed from our study (Advantage V2.1 Assay Kit). While all kits are valid, there are differences in the epitopes used for the detection of analytes across kit versions that may prohibit direct comparison of raw biomarker measurements (27).

### 4.1 Strengths and limitations

Our study has several notable strengths, as well as some limitations. First, our study is among a small collection of studies to investigate plasma biomarkers of neurodegeneration and neuroinflammation in T1D. Our results add knowledge to the emerging field investigating the link between diabetes and neuropathology specifically related to neurodegenerative disease. Our study included an age-matched control group without diabetes and utilized validated, ultra-sensitive assay technology for plasma biomarker quantification. Further, given the extensive data collection within the parent CACTI study, we were able to account for known diabetes-related and insulin-related confounders that may impact the relationships between T1D and neurodegenerative pathology.

Limitations of our study include the relatively small sample size, which consisted of predominantly NHW adults. As previously noted, there was not sufficient representation of other race/ethnicities in this study’s sample to support meaningful comparisons, so race/ethnicity was dichotomized to NHW vs. all others which likely obscures heterogeneity and limits the generalizability of our results. Further, the small sample size significantly limits the sensitivity analysis, where the T1D group was stratified by DR severity. Second, we employed a cross-sectional design and thus cannot make any assertions from our results regarding causality. While these biomarkers have been associated with neuropathology in other populations, the clinical significance of this study’s findings in those with T1D remains uncertain. Accordingly, the present findings should be viewed as evidence of differences in plasma biomarker burden associated with T1D, rather than evidence of active neuropathology or future risk. Our results require validation in longitudinal studies examining relationships before consideration for any clinical application. Third, as previously mentioned, the presence of comorbidities is suggested to alter plasma concentrations, and some of these plasma biomarkers are suggested to relate with other diabetes complications, such as plasma levels of NfL and diabetic polyneuropathy (55). Indeed, GFAP has been suggested to predict retinal neurodysfunction in adults with T2D and early DR (56). In this study, we assessed relationships with DR severity, as well as eGFR as a marker of kidney function, but were unable to include other diabetes complications such as peripheral neuropathy or overt diabetic kidney disease, due to the very low prevalence of these complications in this cohort.

Fourth, APOE genotype status was not assessed for this study. This is a significant limitation, as APOE ϵ4 is an established genetic risk factor for brain amyloid deposition and AD, and is associated with plasma concentrations of AD-related biomarkers, including pTau-181 and Aβ42/40. Consequently, we were unable to assess whether APOE genotype contributed to this study’s findings. Future studies incorporating genetic risk profiling will be necessary to disentangle the relative contributions of T1D-related pathophysiology and underlying AD susceptibility to plasma biomarker levels.

Finally, and perhaps most importantly, this study is limited by the absence of concurrent cognitive and neuroimaging assessments. Although these biomarkers have been shown to be associated neurodegenerative and neuroinflammatory processes in other populations, circulating biomarker levels provide only indirect evidence of underlying neuropathology. Consequently, we cannot determine whether the biomarker relationships observed in adults with T1D are associated with differences in neurocognitive structure/function, and/or increased risk of future neurodegenerative disease. The neurological significance of these plasma biomarkers has not been well characterized in T1D, and therefore the extent to which observed biomarker elevations reflect clinically meaningful relationships remains uncertain.

Although the group of biomarkers analyzed in this study were selected *a priori* based on biological relevance, multiple statistical comparisons were performed across several biomarkers and sensitivity analyses. Formal multiplicity correction was not applied because the analyses were designed to evaluate a targeted set of biologically and statistically correlated biomarkers, rather than to identify associations from a large-scale discovery screen. Nevertheless, the potential for Type I error should be considered when interpreting the findings, particularly those with modest statistical significance. Replication in independent cohorts and longitudinal studies will be important to confirm the observed associations.

## 5 Conclusion

These findings suggest that, in adults, T1D is associated with a greater burden of plasma biomarkers of neurodegeneration and neuroinflammation (pTau-181, GFAP, NfL), and microvascular complications such as retinopathy or impaired kidney function may further contribute to this burden. Further studies, especially longitudinal prospective studies including cognitive testing and brain imaging, are needed to validate our findings and to fully characterize the interplay between these biomarkers and central and peripheral neuropathology in adults with T1D.

## Data Availability

The raw data supporting the conclusions of this article will be made available by the authors, without undue reservation.
