# Supplementary material for: Type 1 diabetes is associated with higher plasma levels of biomarkers of neurodegeneration and neuroinflammation
Source: Front Immunol. 2026 Jul 22;17:1860993. doi: 10.3389/fimmu.2026.1860993 (PMC13437782; doi:10.3389/fimmu.2026.1860993)
Supplement: Supplementary file 1 [file Table1.docx]

Supplementary Material

| **Supplementary Table 1. Plasma biomarker measurements by group.** | | | | |
| --- | --- | --- | --- | --- |
| **Biomarkers** | **Controls**  **n=46** | **All T1D**  **n=90** | **T1D with None/Mild DR**  **n=47** | **T1D with Moderate/Severe DR**  **n=43** |
| **pTau-181 in pg/mL**  **(median [IQR])** | 16.65  [10.23, 20.76] | 17.86  [12.43, 22.23] | 17.01  [12.43, 18.98] | 20.29  [13.97, 24.47] |
| **Aβ42/40 in pg/mL**  **(median [IQR])** | 0.040  [0.036, 0.046] | 0.039  [0.036, 0.044] | 0.039  [0.037, 0.044] | 0.038  [0.036, 0.043] |
| **GFAP in pg/mL**  **(median [IQR])** | 88.30  [73.53, 113.69] | 110.80  [67.36, 156.00] | 103.72  [63.91, 157.55] | 127.23  [90.99, 177.98] |
| **NfL in pg/mL**  **(median [IQR])** | 8.45  [5.91, 12.10] | 10.71  [7.21, 14.73] | 10.34  [6.48, 14.04] | 11.87  [9.01, 16.94] |
| **Tau in pg/mL**  **(median [IQR])** | 3.08  [2.82, 3.67] | 3.44  [2.63, 4.31] | 3.38  [2.50, 4.06] | 3.75  [2.81, 4.81] |
| Data presented as median and interquartile range. Abbreviations: T1D, type 1 diabetes; DR, diabetic retinopathy; picograms per milliliter, pg/mL; IQR, interquartile range; pTau-181, phosphorylated tau-181; Aβ42/40, amyloid beta 42/amyloid beta 40; GFAP, glial fibrillary acidic protein; NfL, neurofibrillary light chain; Tau, total tau; | | | | |
